# Increase of tropical cyclone-induced coastal flooding due to historical sea level rise

**DOI:** 10.1088/1748-9326/ae962b

**Published:** 2026-11-03

**Authors:** Matthias Mengel, Inga Sauer, Christian Otto, Simon Treu, Lisa Novak, Katja Frieler

**Affiliations:** 1 Potsdam Institute for Climate Impact Research (PIK), Member of the Leibniz Association, PO Box 60 12 03, D-14412 Potsdam, Germany; 2 Department of Environmental Science and Geography, University of Potsdam, Potsdam, Germany

**Keywords:** sea level rise, coastal flooding, tropical cyclones, surges, attribution

## Abstract

Surges from tropical cyclones (TCs) cause significant damage in coastal areas. However, the contribution of historical sea level rise (SLR) to surge heights and thus the flooded areas has not been quantified on a global scale. Here, we use an open-source shallow water equation solver to estimate the impact of historical SLR on coastal flooding induced by all historical TCs with landfall from 1950 to 2025, according to the International Best Tracks Archive. We isolate the SLR effect through the comparison of factual simulations accounting for observed changes in sea level to counterfactual simulations without long term trends in sea levels. Results show that SLR has steadily increased the coastal area affected by TC-induced surges, from about 5% in 1950–1954 assuming mean tide levels (7% and 5% for minimum and maximum tide levels, respectively) to about 18% in 2021–2025 (22% and 13% for minimum and maximum tide levels, respectively) in comparison to the areas affected without SLR. The increase differs along coastlines, with maxima in relative increases of surge flooding along the North American Atlantic coastline and the Southeast Asian coastline. To put the numbers into perspective, we also estimate the associated increases assuming 1 m of local SLR and no changes in TC tracks and wind fields, reaching 146% under mean tide assumptions (192% for minimum tide levels and 92% for maximum tide levels). Results show that this is higher than expected assuming a linear relationship between SLR and relative increases in flood extents. Our analysis demonstrates the significant increase in flood extent induced by SLR already today. The generated data sets of factual and counterfactual coastal flooding extend ISIMIP3a climate-related forcing data, allowing for the evaluation and attribution of additional coastal impacts.

## 1. Introduction

Tropical cyclones (TCs) are the most destructive weather extreme, responsible for 44.3% of global direct economic losses, 25.8% of fatalities induced by weather-related disasters reported by EM-DAT over 2000–2025 (Delforge *et al* 2024) and 27.6% of the total number of people newly displaced by weather-related disasters reported by the IDMC since 2008 (Internal Displacement Monitoring Centre 2023).

While it is likely that the global proportion of major (Category 3–5 on the Saffir–Simpson scale) cyclones has increased over the past four decades (Seneviratne *et al* 2023, Masson-Delmotte *et al* 2023, Seneviratne *et al* 2023) there is still ‘low confidence in long-term (multi-decadal to centennial) trends in the frequency of all-category TCs’ (Masson-Delmotte *et al* 2023). However, there is growing evidence that climate change has increased precipitation associated with TCs and that land–sea contrasts may further enhance rainfall rates of TCs during landfall (Guzman and Jiang 2021, Tu *et al* 2022, Zhong *et al* 2026a, 2026b).

In addition, global mean sea level increased by 0.20 m (IPCC ‘very likely’ range (90% interval): 0.15–0.25) between 1901 and 2018 (Masson-Delmotte *et al* 2023) with human influence being the main driver of increasing sea levels since at least 1971 (Masson-Delmotte *et al* 2023, Dangendorf *et al* 2025). Meanwhile, the rate of global sea level rise (SLR) has reached 4 mm per year in 2019 leading to an additional 0.02 m of SLR from 2019 to 2024 (Dangendorf *et al* 2024). Both rising sea levels and increases in TC-induced precipitation are expected to increase TC-induced flooding. However, the contribution of these drivers to historical TC-induced flooding has not been quantified on a global scale. In our study, we focus on the contribution of SLR, but ignore rain-induced flooding.

So far global modelling studies have focused on the simulation of extreme coastal water levels (Muis *et al* 2016), with recent improvements in the representation of TC-related water levels (Muis *et al* 2020). In addition, a bathtub approach has been used to estimate historical surge affected areas for all storms of the IBTrACS archive (Knapp *et al* 2010) based on simulated coastal water levels by the hydrodynamic Delft3D model (Kunze and Strobl 2024). However, these global studies do not account for the effects of historical long-term SLR on coastal water levels. Other hydrodynamic models allowing for the assessment of surge-inundated areas have only been applied to a small set of individual events (Bates *et al* 2021, Liang and Zhou 2022, Wijetunge and Neluwala 2023, Eilander *et al* 2023b).

Here, we use the dynamical flood modelling chain of (Vogt *et al* 2024) to provide first global estimates for the effects of rising sea levels on TC-induced coastal flooding. The approach builds on the dynamic shallow-water equation solver GeoClaw (Berger *et al* 2011, Mandli and Dawson 2014) which calculates storm surge inundation from wind forcing over both ocean and potentially flooded land areas. The dynamic one-step approach overcomes the simplified bathtub assumption applied in common two-step approaches separating the water level simulation from the estimation of the flood extent (Sampson *et al* 2015, Muis *et al* 2016). As a consequence of the dynamic representation of inundation processes, the GeoClaw flood extents agree better with satellite observations compared to usually applied bathtub approaches (Vogt *et al* 2024).

The model is forced by the observed TC record, combined with observational factual geocentric sea level data and associated counterfactual data where long-term trends have been removed (Treu *et al* 2024) (see Methods). This setup allows us to explore the influence of SLR while keeping observed storm tracks and wind fields unchanged. While tide levels vary along TC tracks, our simulations only use a constant tidal offset representing either the minimum (min), mean, or maximum (max) tide level that occurred at the centre of the model domain of the storm in the year of the storm. Assuming no temporal correlation between the occurrence of the TC and tide levels, we consider the mean tide assumption the best estimate and use it as the main specification but also provide calculations for the min and max tide assumptions.

Modelling only storm surge, the approach is not designed to exactly reproduce individual flood events that also depend on rain-induced flooding as well as on the dynamic interaction between tides and surge not represented in GeoClaw. However, it allows us to examine whether, and to what extent, SLR has increased the annual area affected by TC-induced storm surges globally over the historical period and whether the contribution of SLR to storm surge exposure has increased over time. To put our historical estimates into perspective, we also estimate relative increase in flooding induced by 1 m of local SLR in comparison to the counterfactual ‘no SLR’ assumption.

## 2. Methods and data

### 2.1. Storm surge simulations

We adopt and update the TC surge modelling setup based on GeoClaw of (Vogt *et al* 2024). GeoClaw includes the relevant physical processes for the intended global assessment: wind and pressure forcing from a parametric TC model, wind drag and bottom friction, depth-averaged shallow-water equations for storm surge flow accounting for SLR at the time and coastal location of the storm in the factual simulations (see section 1 of supplementary information (SI) for a detailed model description). In contrast to the previous setup, we use the monthly sea level reconstruction from Treu *et al* (2024)the long-term quadratic trend since 1900 (‘no SLR’ counterfactual). For each storm, we select the factual and counterfactual sea level from the month in which the storm made landfall.

To describe the water levels as the height of the ocean’s surface relative to a specific piece of land, we combine different data sets to finally reference elevation, bathymetry, and water levels to the geoid model GOCO06s (Kvas *et al* 2021). The common referencing ensures vertical datum consistency to overcome the problem of inconsistent reference systems, which has been identified as an important source of bias in many coastal impact studies (Seeger and Minderhoud 2026).

The potential effects of tides are represented by a uniform offset applied to the entire model domain, calculated as the deviation of the min, mean, or max astronomical tide from the annual mean tide level by the FES2014 tidal model (Lyard *et al* 2021)at the centre of the model domain for the year in which the storm made landfall.

To prescribe TC characteristics 6-hourly observational information about the location of the storm centre, central pressure, maximum 1 min sustained wind speed, environmental pressure, radius of maximum wind speeds and the radius of the outermost closed isobar from version v04r01 of the IBTrACS dataset (Anon 2024) are used as input for a parametric wind and pressure field model (Holland 1980) which generates the forcing for the calculation of the storm surge-affected areas with the open-source geophysical flow solver GeoClaw (Berger *et al* 2011, Mandli and Dawson 2014) GeoClaw solves the depth-averaged shallow water equations in a regional domain that covers both ocean and potentially flooded land area. The dynamic one-step approach overcomes the separation of models between ocean and land, which is still common in surge modelling (Sampson *et al* 2015, Muis *et al* 2016). GeoClaw enables a seamless simulation of storm surge inundation from wind forcing at manageable computational cost, even for large sets of storms. From the 8394 storms recorded in IBTrACS for the period 1950–2025, 4770 are included in our study as they cause coastal flooding in the factual or in the counterfactual run (supplementary figure S3). As in Vogt *et al* (2024), all simulations are performed at 30 arc seconds (approximately 1 km) resolution.

Geoclaw computes a flood depth time series for each grid cell within the modelling domain, including both ocean and land cells. To obtain flooded areas on land, we use the maximum depth over time and first mask out all grid cells where max flood depths <0.1 m. In addition, we use the Copernicus Global Surface Water Occurrence dataset (Pekel *et al* 2016) in version 1.1, which covers the period 1984–2019, derived from Landsat imagery at 30 m spatial resolution to mask out permanent water bodies. Thereby, we assume that a grid cell is a permanent water body if it was flooded for more than 5% of the observational period. We interpolate the permanent water body data to 30 arc seconds to match the grid of our flood simulations. Finally, we exclude areas that lie higher than 10 m above sea level in the digital elevation data. For each storm, this yields the factual and counterfactual binary flood maps on the model domain. To compare the simulated data to the observational flood extent from Dartmouth Flood Observatory (DFO) (Kettner *et al* 2021) (figure 1), the RAdar-Produced Inundation Diary (RAPID) (Shen *et al* 2019), and the Global Flood Database (GFD) (Tellman *et al* 2021) (supplementary figure S5–S80). The observational data is upscaled to the coarser simulation grid. To this end, we use a maximum-within-grid-cell approach, i.e. we assign each coarse-grid cell the maximum value of all fine-grid source pixels that fall into it. This approach ensures that a coarse cell is marked as flooded whenever at least one contributing fine-resolution pixel is flooded.

**Figure 1.**
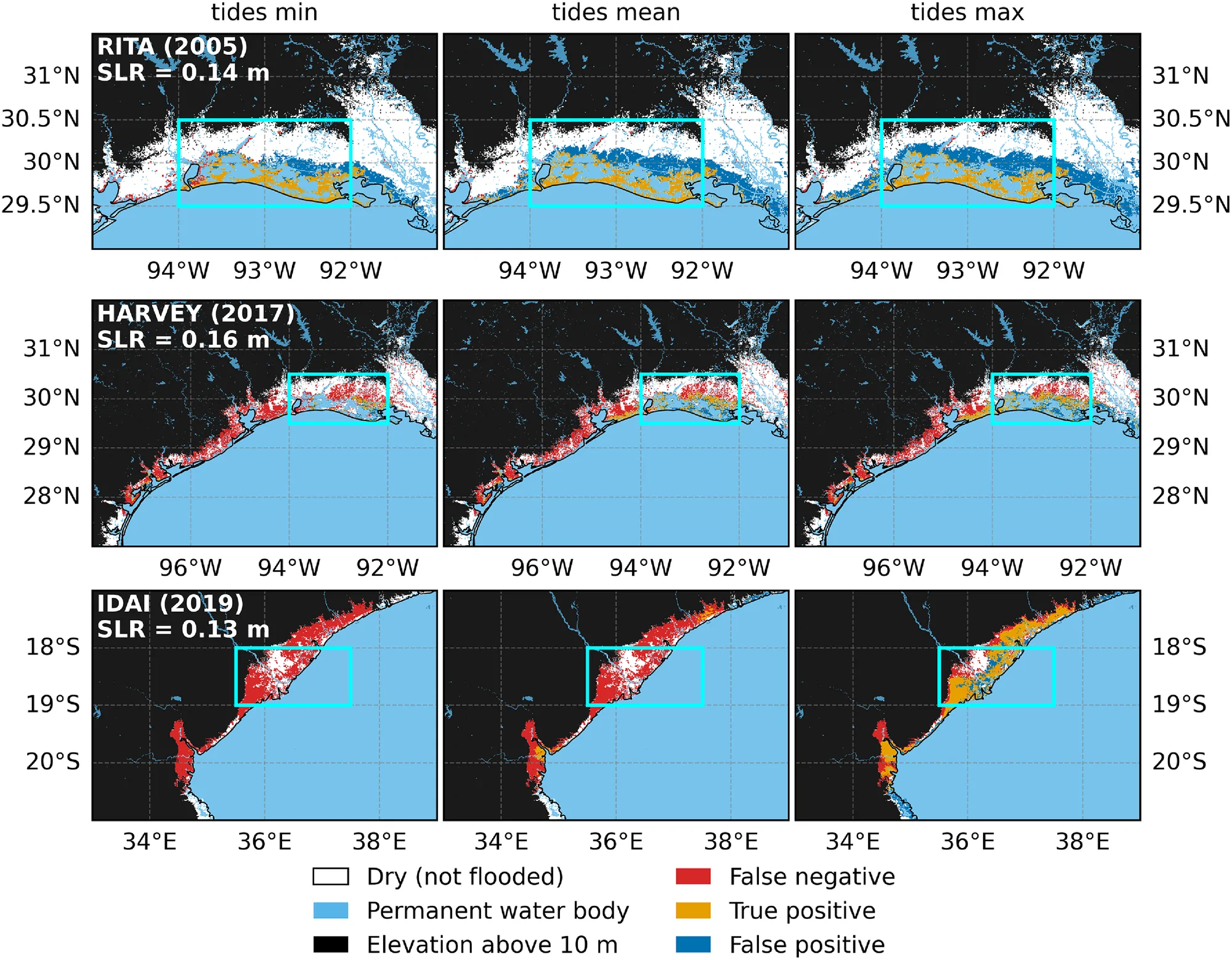
Illustrative comparison of simulated surge areas to observed total flood extent for three major surge events (different rows), based on minimum tide, mean tide and maximum tide assumptions (different columns). Surge flooding does not necessarily need to match the total observed flood extent, which is a combination of surge, river and rain flooding. Observed flood extent from the Dartmouth Flood Observatory (Kettner *et al* 2021). The indicated cyan rectangles represent the areas we zoom in when showing the difference between the factual and counterfactual flood extent in figure 2.

The factual and counterfactual GeoClaw simulation data are part of the climate-related forcing (‘counterclim’) used as input for the impact model evaluation and impact attribution tasks within the third round of the Inter-Sectoral Impact Model Intercomparison Project (ISIMIP3a, Frieler *et al* 2024). For the ‘1 m SLR’ scenario, we add, for each storm, 1 m of SLR to the local sea level in the counterfactual without SLR and calculate the resulting storm surge.

### 2.2. Data analysis

#### 2.2.1. Changes in inundated areas due to SLR

To estimate the change in flood extent that is induced by SLR, for each storm the flooded grid cells over land are aggregated to derive the flooded land area for the factual simulations with SLR (*A*_SLR_) and its counterpart from the counterfactual simulation without SLR (*A*_noSLR_) per storm. For global time series (figure 3), we sum up the event areas per year to yield global yearly factual and counterfactual time series. For aggregation along coastlines (figure 4), we first produce global annual maps that indicate how often a grid cell was flooded and then sum over years of interest (whole coverage 1950–2025 and last decade 2016–2025). Finally, the factual and counterfactual frequency maps are multiplied by the areas of the grid cells and flooded areas are aggregated within circles with a radius of 2° moving along the impacted coastlines (supplementary figure S77) taken from Natural Earth (2025).

From the difference of flooded areas with and without SLR (*A*_SLR_—*A*_noSLR_) the relative fraction of land area flooded due to SLR is calculated as Δ*A*_frac_ = (*A*_SLR_—*A*_noSLR_)/*A*_noSLR_. For global time series, the relative changes are calculated from the annually aggregated factual and counterfactual data and not from an aggregation of the storm specific relative changes. For the data along coastlines, relative changes are calculated for each circle using the temporally and spatially aggregated data (1950–2025 in figure 3 and 2016–2025 in figure 4). Our metric Δ*A*_frac_ differs from the flooded area that is attributable to SLR defined as Δ*A*_attr_ = (*A*_SLR_—*A*_noSLR_)/*A*_SLR_, which is the analogue to the fraction of attributable risk (FAR) used within climate attribution science. The latter is defined as FAR = (P_1_–P_0_)/P_1_ where P_1_ is the probability of an event of a certain intensity under factual climate forcing while P_0_ is the probability in a counterfactual world without anthropogenic climate forcing (Otto 2023). While the increase in the ‘fraction of flood extent attributable to SLR’ is bounded by unity, the ‘relative increase in flood extent due to SLR (Δ*A*_frac_)’ can exceed unity.

To assess potential influences of physical drivers, such as translation speed, minimum central pressure as a proxy for storm intensity, and SLR, we apply a quantile regression to evaluate the extent to which these variables explain the variability in the relative increase in flood extent at the individual storm level. To derive the translation speed of storms, we extract the locations of storm centres over the simulation period from IBTrACs over time and calculate the average speed as distance per time. Similarly, we average the recorded central pressure values over the GeoClaw simulation period of each storm. SLR is incorporated in the regression analysis using the difference in sea level between the counterfactual and the factual realization of the storm surge.

We also test to what degree the absolute and relative increase in flooding due to SLR can be approximated and extrapolated in terms of SLR. To this end, we use linear regressions to statistically link the global annual absolute and relative increases in flood extent to SLR. The latter is defined as the annual average across all TC-specific SLR data (= factual—counterfactual sea levels). The model is fitted to the historical factual and counterfactual annual data and tested to predict the absolute and relative increase in flood extent induced by 1 m of SLR.

## 3. Results

### 3.1. Model characteristics and performance

The correspondence of the modelled flooded areas with those obtained from satellite imagery is quite mixed. For an illustrative comparison of modelled and DFO flooded areas, we have selected Hurricane Rita (2005) and Hurricane Harvey (2017), which both made landfall at the Caribbean and Gulf of Mexico coastlines as well as TC Idai (2019), which made landfall in Mozambique as these examples illustrate both substantial overestimation and underestimation of the observed flood extent, as well as the sensitivity of the results to tidal assumptions. The three cases allow for a discussion of the potential reasons for the variation of outcomes. Results for all other TCs which can be matched to satellite observations from DFO, GFD, or RAPID are shown in the supplementary figures S1–S76.

As the model setup only captures the coastal surge component of the total flooding induced by TCs, which usually also comprises rain-induced flooding (Eilander *et al* 2023b), our simulations are expected to generally underestimate observed flooding, as is the case for Hurricane Harvey and Idai (figure 1). On the other hand, we do not account for potential flood protection by, e.g. dykes which could produce larger simulated flood extents than in the observations.

The underestimation of flood extent for Hurricane Harvey is very likely associated with significant rain-induced flooding not captured by GeoClaw: Dedicated assessments of Hurricane Harvey show that the flooding was mainly induced by rainfall and consequent river flooding (Lee *et al* 2024). For Idai the observed flood extent can be well reproduced under max tide assumption. However, Idai occurred close to neap tide, and flooding of the Buzi and Pungwe rivers contributed substantially to the overall flooding (Eilander *et al* 2023a, Vertegaal *et al* 2025).

Despite missing the rain-induced flooding, GeoClaw overestimates the flooding induced by Hurricane Rita (figure 1, upper row) even under minimum tide simulations, indicating that other reasons than tides are responsible for the mismatch. The overestimation could be due to deficits in GeoClaw in capturing the specific characteristics of the event or missing representation of flood protection, but could also be associated with a general tendency of satellite‐derived inundation estimates to underestimate the true maximum flood extent as (i) satellites do not necessarily visit the affected area during the period of peak flooding, or (ii) cloud cover may obstruct the detection of surface water (Vogt *et al* 2024) for a detailed comparison of the flooded areas as provided by the different satellite-based products).

The different potential sources of the mismatch between the simulated and observed flood extents make it difficult to evaluate the simulations and to determine the contribution of SLR to individual observed flood extents. That is why, in this paper, we provide the overall global numbers derived from the GeoClaw simulations. Due to its ability to capture inundation processes dynamically, we consider this modelling setup well suited to assess the average contribution of SLR to storm-surge–affected areas across multiple TCs.

### 3.2. Increase in flood extent due to SLR

The areas flooded due to SLR (purple areas in figure 2) typically appear as a narrow, spatially sparse band along the outer margin of the total inundation area. There are also small areas that are flooded in the simulations without SLR but stay dry in those with SLR (e.g. green areas near 94° longitude for Harvey and mean tide in figure 2). This may occur because of complex dynamic interactions captured by the two-dimensional shallow water equations (LeVeque *et al* 2011). However, the areas where factual water depths are smaller than the counterfactual depths are limited to less than 0.01% of the total factual global flood extent shown in figure 3(B).

**Figure 2.**
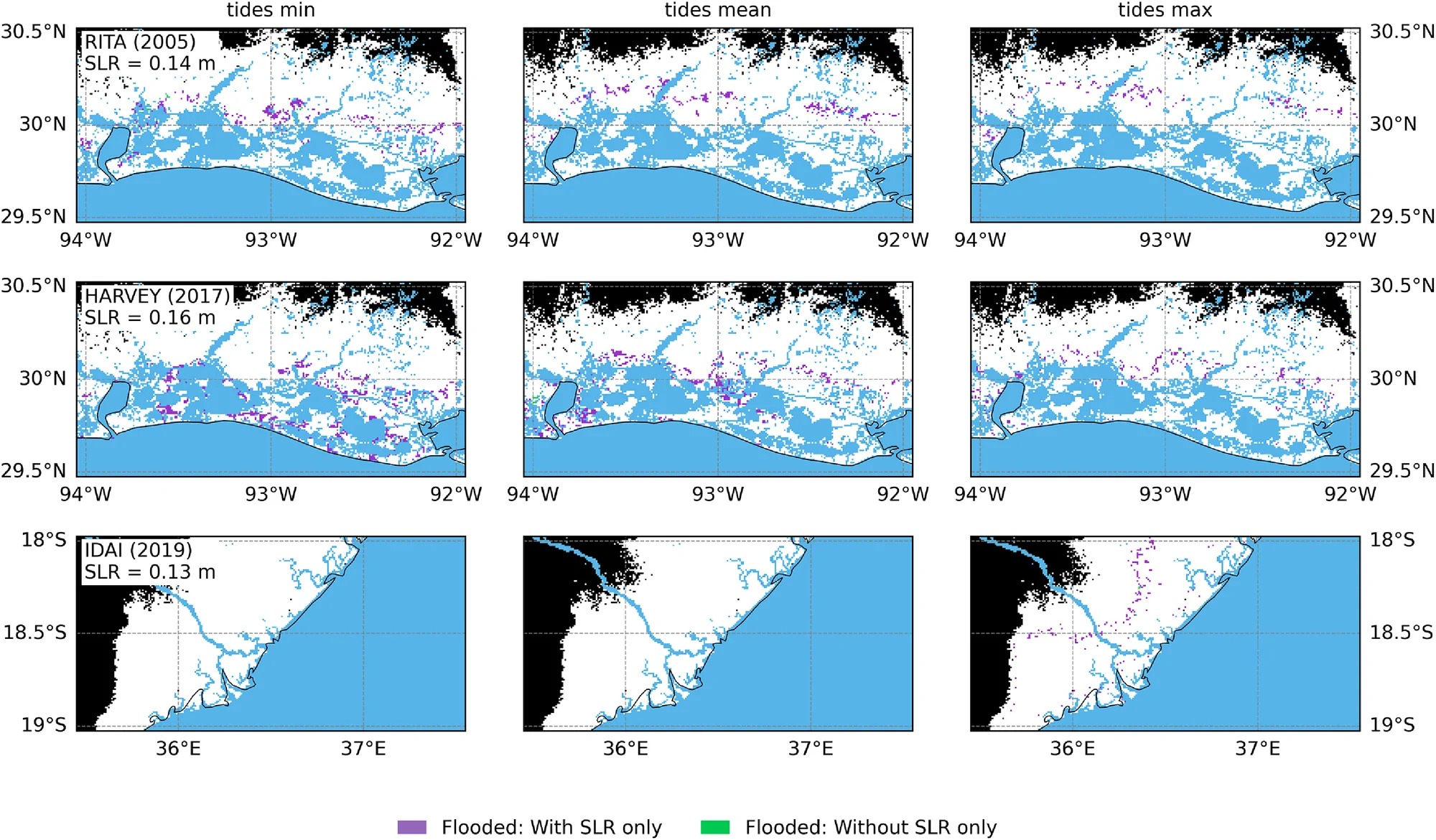
Comparison of simulations with and without SLR. Pixels that are flooded in the factual simulations accounting for long-term sea level rise but not in the counterfactual simulations without long-term SLR (purple); pixels that are flooded in the factual simulations, but not in the counterfactual simulations (green). Pixels where factual and counterfactual simulations agree in indicating flooding or no-flooding are shown in white. Permanent water bodies shown in blue, land areas with elevations >10 m shown in black. Results are shown for the same simulations as in figure 1 and the sub-regions indicated there.

For the three illustrative examples, the areas flooded due to SLR are still relatively small compared to the total flooded areas (comparison of purple areas in figure 2 to orange + dark blue areas in figure 1) in line with the global estimates covering around 4770 storms from 1950 to 2025 (figure 3(B)).

This is expected as the surge height of flood events with relevant flooded areas is much larger than SLR, so SLR is not the dominant driver of total flooded area of individual flood events. However, its effect is increasing over time with rising sea levels, both in absolute terms (figure 3(C)) and relative terms (figure 3(D)).

**Figure 3.**
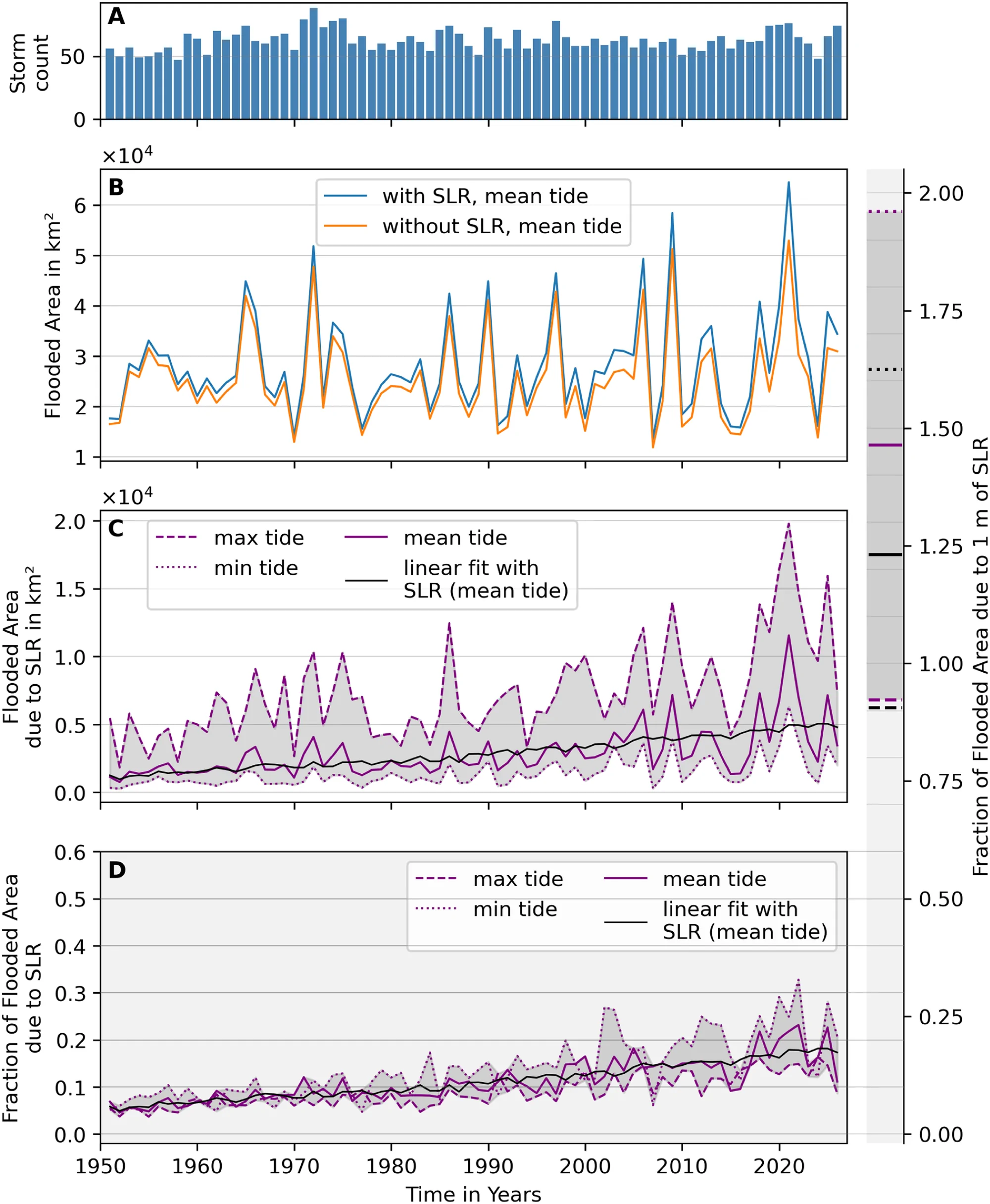
Globally aggregated increase of TC-induced flooding due to sea level rise. Panel A: Number of storms per year leading to flooding in the factual or counterfactual simulations. Panel B: Time series of factual (blue line, accounting for observed sea level rise) and counterfactual (orange line, assuming no long-term sea level rise) globally aggregated annual TC-induced coastal flooding under mean-tide assumptions. Panel C: Absolute increase in flooding due to sea level rise (= difference between factual and counterfactual global flood simulations) under mean tide (solid purple line), maximum tide (max, dashed purple line), and minimum tide assumption (min, dotted purple line) The solid black line shows the regression results using average annual sea level rise across the events as predictor. Panel D: Relative increase in flood extent compared to counterfactual areas (Δ*A*_frac_), purple line types as in panel C. Right part of panel D: Δ*A*_frac_ for simulations assuming 1 m SLR and min (purple dotted line), mean (purple solid line) and max (purple dashed line) tides, respectively. Predictions from linear regression on the historical 1950–2024 data on sea level rise are shown in black (same line style for the different tides).

In principle, changes in *A*_SLR_ and *A*_noSLR_ over time could result from reporting biases. For instance, the fraction of storms not reported in IBTrACS could decrease over time. However, this should bias factual and counterfactual flood extent in the same way. Thus, biases should already be reduced when considering the absolute difference between factual and counterfactual flood extents, *A*_SLR_—*A*_noSLR_ and they should generally cancel out for relative differences Δ*A*_frac_.

In our case, the annual number of storms considered in the calculation of *A*_SLR_ and *A*_noSLR_ does not show a trend (figure 3(A)). Thus, potential reporting biases can neither explain the changes in *A*_SLR_ and *A*_noSLR_ over time (figure 3(B)) nor the observed trends in *A*_SLR_—*A*_noSLR_ (figure 3(C)) and Δ*A*_frac_ (figure 3(D)). In addition, the year-to-year variability in flooded areas (figure 3(B)) is only partly explained by the number of storms per year (*R*^2^ = 29%). The explanatory power of storm frequency decreases for the flooded area attributable to SLR (figure 3(C), *R*^2^ = 15%), and decreases further for Δ*A*_frac_ (figure 3(D), *R*^2^ = 3%), suggesting that Δ*A*_frac_ is a metric unaffected by annual storm frequencies and potential reporting biases in storm numbers.

While the absolute difference between the factual and counterfactual global flooded area strongly depends on tide assumptions, with the difference being systematically the largest under max tide assumptions and smallest under min tide assumptions (figure 3(C)). In contrast, tide assumptions are less relevant for Δ*A*_frac_. Relative effects of SLR are generally strongest under min tide assumptions and lowest under max tide assumptions, with relative changes under min tide assumptions sometimes falling below the differences under max or mean tide assumptions (figure 3(C)). This indicates that the relative increase in the global annually affected area does not critically depend upon the tide assumptions. With increasing sea level, Δ*A*_frac_ increases from about 5% in 1950–1954%–18% in 2021–2025, while a 1 m SLR roughly doubles to triples the counterfactual flood extent assuming no SLR (Δ*A*_frac_ = 1.46 under mean tide assumptions, 1.96 under min tide assumptions, and 0.92 under max tide assumption, respectively; right part figure 3(D)). The linear regression on annual SLR well reproduces the long-term increase in the absolute and relative changes in flood extent induced by SLR over the historical period (see black lines in figures 3(B) and (C)). The increase in flooding induced by SLR increases with roughly 3800 km^2^ per 10 cm of SLR. At the current rate of SLR of 4.5 mm yr^−1^ (Hamlington *et al* 2024), the area flooded due to SLR from TC surges increases by about 172 km^2^ per year. However, the regression model strongly underestimates the increases in flooded extent induced by 1 m of SLR at least under the min and mean tide assumption (see difference between the purple (process-based projections) and black (empirical projections) dotted and dashed lines in the right part of figure 3(D)) indicating that the assumption of a linear relationship between SLR and increase in TC-induced coastal flooding represents an oversimplification. While covering the long-term trend, shorter term fluctuations in flooded areas cannot be predicted by the linear regression on SLR. They may result from fluctuations in TC landfall positions. Testing other physical characteristics of the TC as predictors of Δ*A*_frac_ shows that minimum central pressure (as a proxy for storm intensity) and translational speed are only insignificantly related to TC specific Δ*A*_frac_ (*p*-value_min pressure_= 0.31; *p*-value_translation speed_ = 0.43). These characteristics have already changed due to global warming (Kossin 2018, Knutson *et al* 2019) and are expected to further change with additional warming (Knutson *et al* 2020, Yamaguchi *et al* 2020). Yet, over the historical period analysed in this study, SLR is the only significant predictor for TC specific Δ*A*_frac_ (*p*-value_SLR_ < 0.01).

TC flooding is a local phenomenon, which is even more true for the surge component of the flooding, which is shaped by the combination of storm characteristics, coastal geometry, bathymetry, and tidal conditions at landfall. Storm surge-induced flooding and its amplification by SLR are therefore not distributed evenly along global coastlines, but strongly concentrated in specific regions in the tropical and subtropical latitude bands (roughly between 5° and 30° in both hemispheres, figure 4).

**Figure 4.**
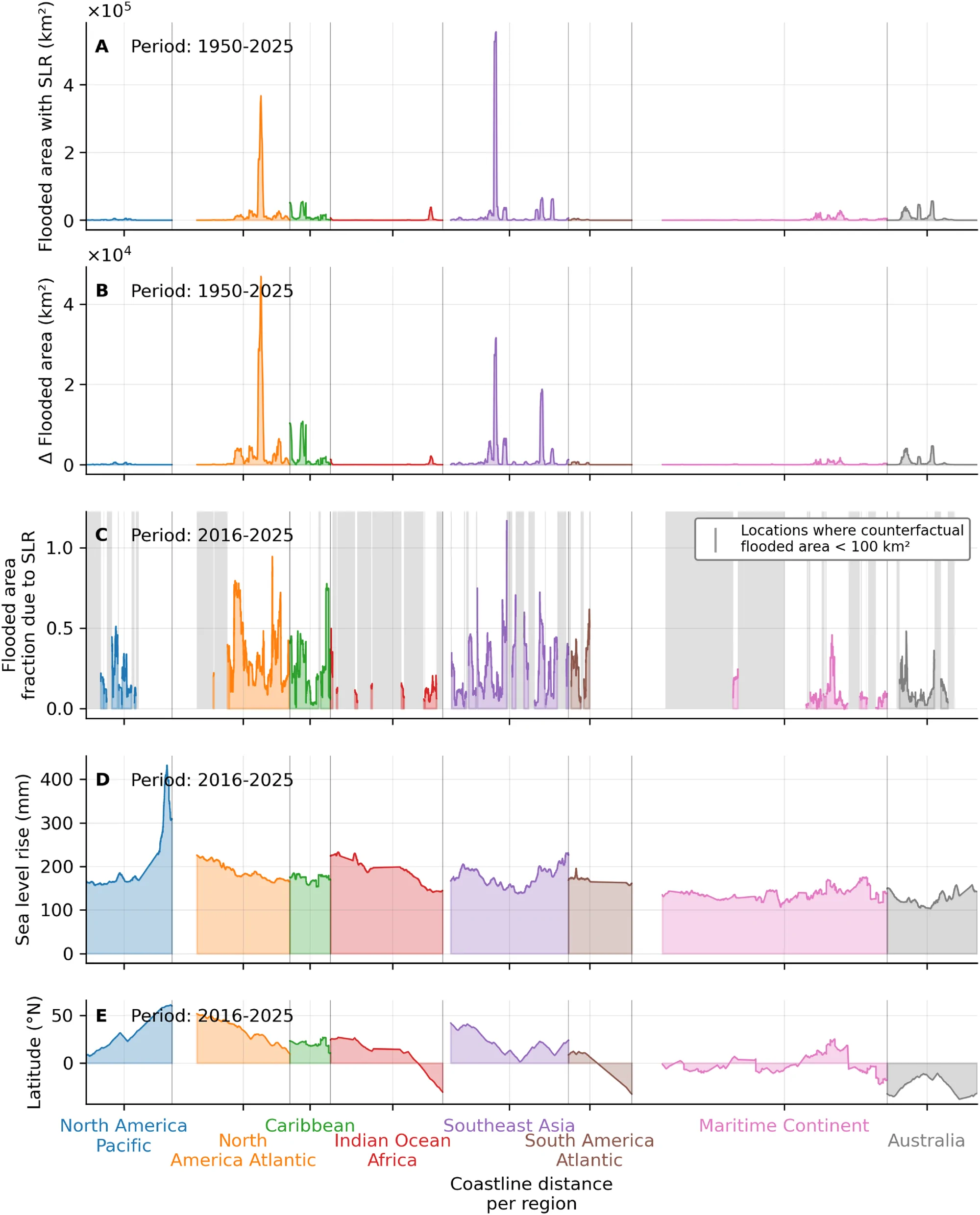
Spatial distribution of TC-induced coastal flooding and historical contributions of sea level rise. Panel A: Simulated flooded areas with mean tide assumptions and historical sea level rise, accumulated from 1950 to 2024. Panel B: Difference between simulated areas with and without sea level rise, accumulated from 1950 to 2024. Panel C: Fraction of flooded area from sea level rise in the period 2016–2025. Points with counterfactual areas *A*_noSLR_ < 100 km^2^ are excluded to avoid peaks due to division by small numbers. The missing points are indicated by grey vertical lines which in sum appear as shading if neighbouring points are excluded. Figure S4 in the SI shows the same results for different cutoff values. Panel D: Local sea level rise in 2016–2025 compared to the 1900–1905 average (see figure S2 and associated text for details). Panel E: Latitudes of the different data points along the coastlines (see figure S1 for definition).

On the disaggregated local level, the total area flooded by TC surges from 1950 to 2025 can be strongly influenced by the TC frequency, i.e. the total number of storms per year. In addition, the spatial distribution can be influenced by the topography and the geometry. It is dominated by two peaks in the Gulf of Mexico region (North America Atlantic) and Southeast Asia. The cumulative absolute increase in flood extent due to SLR (figure 4(B)) mimics the shape of the absolute extent (figure 4(A)). This correspondence suggests that the local magnitude of the SLR effect scales with overall surge exposure so that regions that are flooded often or extensively under current conditions gain the most additional area when SLRs.

In contrast to the absolute differences, the fractions of the area flooded to SLR is no longer dominated by the frequency of the events as normalized by the counterfactual cumulative flood extents and shows a more even distribution along global coastlines, with most values between 0.1 and 0.4, implying that SLR is typically responsible for a 10%–40% increase in surge-flooded areas compared to a situation without SLR. In contrast to the globally aggregated numbers, the spatially resolved relative increases in the flooded areas can be affected by very small counterfactual areas, inducing peaks in the relative changes. To avoid associated peaks in the data we do not show the relative increase in flood extent due to SLR where associated counterfactual flood extent within the given circle and time span (2016–2025) is smaller than 100 km^2^. Except for individual spikes, results are relatively insensitive to the choice of the threshold (figure S4 in the SI). Overall, the pattern of relative increases in flooding does not seem to be connected to spatial variations in SLR (figure 4(D)).

## 4. Discussion

In this work, we combined recordings of TC tracks with the dynamics shallow-water equation solver GeoClaw to quantify the increase in storm surge affected areas due to SLR for a large, global set of 4770 historical storms over the period 1950–2025. This allows us to assess global SLR-induced trends in TC storm surge–affected areas, showing that the contribution of SLR to TC-induced coastal flooding has increased over time, from about 5% in the 1950s to 18% in the last decade (2016–2025). In this regard, we go beyond the existing literature on attributing TC-induced flooding, which so far has focused on individual events such as Hurricane Harvey (Smiley *et al* 2022) or TC Idai (Mester *et al* 2023, Vertegaal *et al* 2025). Under ongoing climate change, TC-induced storm surges may not only be intensified by SLR but also by other drivers such as intensity and translation speed. The number of very intense storms has increased over the last decades (Knutson *et al* 2019) and is projected to increase in the future (Knutson *et al* 2021), which may also intensify coastal flooding. Effects of changes in translation speed on storm surges strongly depend on the coastal morphology (Islam and Takagi 2021). The translation speed of TCs has slowed down over the period 1949–2016 (Kossin 2018) under global warming, where sign and magnitude of changes vary strongly across different latitudes (Yamaguchi *et al* 2020). All in all, changes in physical drivers and their effects on TC impacts are complex and show strong regional variations. Our process-based modelling framework allows us to account for changes in intensity and translational speed in the historical period through the recordings of minimum central pressure (as proxy for storm intensity) and translational speed in the IBTrACS data. While intensities and translation speeds only have a minor influence on TC specific Δ*A*_frac_, future changes in landfall positions could have larger effects on Δ*A*_frac_ not accounted for in our estimates of global Δ*A*_frac_ under 1 m of SLR.

However, relying on IBTrACS comes with some limitations, as it is not a reanalysis attempting to provide a homogeneous record over time. It provides a collation of best-track data from different agencies. As such, it may be affected by regional and temporal reporting biases. While theoretically positive trends in the factual and counterfactual flooded areas (figure 3(B)) and also in absolute differences (figure 3(C)) could be induced by a growing number of reported storms due to improved monitoring systems (e.g. transition into the satellite-era), this is apparently not the case here. The number of TCs included in our analysis does not show a trend. Further, the annual changes of this number do not seem to be related to the strong annual variations in flood extent and the absolute attributable flood extent. In addition, the relative increases in flood extent compared to the counterfactual extents should be relatively independent of potentially missed storms.

In contrast to the IBTrACS data, new reanalysis products such as ERA5 covering the period from 1940 to today (Soci *et al* 2024), may provide a more comprehensive figure of historical variations in storm characteristics. While our results are not sensitive to the total number of included storms, inaccuracies in simulated flooded areas may arise through the simulation of symmetric wind fields. This could be overcome by using the more homogeneous ERA5 data. The latest product seems to be less affected by a switch from assimilating IBTrACS data (1940–1978) to not assimilating this data (1979-today) than the earlier product (Bell *et al* 2021). However, there seems to be a general bias in the minimum ERA5 pressure closest to the IBTrACS tracks with ERA5 minimal pressures overestimating IBTrACS minimal pressures (Soci *et al* 2024). There are also existing data products providing improved windfield estimates based on ERA5, yet for the scope of our study they are inadequate due to the short temporal coverage (1993–2022), as improvements, e.g. corrections of the storm centre, also rely on satellite-based input data (Liu *et al* 2025).

Our findings can help inform decision-makers at different levels, such as regional or national adaptation planners charged with the implementation of National adaptation plans or the damage modelling of insurance companies. On the international level, our findings inform the upcoming second Global Stocktake, which, as a key instrument for implementing the Paris Agreement, relies on robust information about climate change impacts. Further, our modelling can provide decision support for the UNFCCC’s Loss and Damage process (Mechler *et al* 2018), in which decision-makers are tasked with allocating funds to compensate strongly affected developing countries for climate-related losses and damages and to support adaptation. For comparing climate-change induced risks across countries, it may be more robust to assess how climate change has contributed to TC impacts over the historical period, rather than focusing on the attribution of individual events.

The employed modelling setup has several limitations. First, the GeoClaw simulations do not represent the rain-induced flooding extending the surge induced flooding associated with TCs. Physical understanding and a growing set of event-specific attribution studies indicate that warming-related increases in heavy precipitation induced by TCs have already extended the TC-induced flooding over the historical period and therefore amplified the positive SLR-induced trends identified in this study. However, while there are modelling approaches that also capture the rain induced fluvial or pluvial flooding (Eilander *et al* 2023b), they are still too computational expensive to be applied on a global scale to thousands of TCs. Ongoing efforts to overcome these limitations (Benito *et al* 2025) may allow for an inclusion of all relevant drivers in future global studies.

In the counterfactual simulations, we do not change the dynamical characteristics of the TCs (storm track and wind speeds) either, i.e. we only quantify the effects of SLR given the observed tracks and wind speeds. Further, SLR is defined as long-term trends in the historical observational data and does not distinguish the effects of recent climate change or anthropogenic climate forcing from delayed responses to the warming after the last ice-age.

Further, our factual simulations are not expected to reproduce each TC-induced flooding perfectly. In addition to the expected systematic bias due to the missing rain-induced flooding, differences between observed and modelled flood extents reflect physical complexity and numerical limitations inherent in GeoClaw that e.g. does not represent the temporal evolution of tides over the course of the events. These imperfections should however not undermine a broader attribution analysis. The model captures the governing physics of surge generation and coastal inundation (Vogt *et al* 2024). While errors at the level of individual storms are inevitable, we expect them to be largely random. Over the large ensemble of 4770 simulated TCs, the random event-scale deviations should average out, whereas the systematic physical signal of sea-level rise should be retained.

The model simulations do not represent the effects of protection measures such as dykes. These measures may counteract the simulated increase in TC-induced flooding as simulated by GeoClaw. In principle, such measures could be implemented in the simulations in a simplified way by assuming no flooding as long as water levels stay below a given protection level. However, the information about protection levels is still very fragmented and does not allow for an assessment of temporal changes. While there are promising approaches to estimate the evolution of effective protection levels from observed damage data (Paprotny *et al* 2024) they are still limited to Europe. The analysis also shows that the estimates reported at the global scale (FLOPROS, Scussolini *et al* 2016) may be higher than the effective levels derived from observed damages.

The present study uses geocentric (absolute) sea level as the background water level in GeoClaw, in combination with a static digital elevation model. This framing captures the effect of ocean water rise on storm surge inundation but does not account for vertical land motion, which in many coastal regions contributes to or even exceeds the geocentric sea level trend. A natural extension of this work would replace the geocentric sea level anomaly with a relative sea level anomaly, referenced to the same period as the DEM.

Assuming no changes in dynamical storm characteristics, the model could be used to estimate the functional relationship between SLR and increases in TC-induced coastal flooding. While our analysis only indicates that the relationship is non-linear, simulations assuming small step increases in sea levels from today’s levels to 1 m or more could help to identify the actual relationship. This may help to account for the effects of SLR in future projections of TC-induced damages on a global scale.

## Data Availability

The data that support the findings of this study are openly available at the following URL/DOI: https://data.isimip.org/10.48364/ISIMIP.693793.1 (Mengel 2026). Supplementary information available at: https://doi.org/10.1088/1748-9326/ae962b/data1.

## References

[erlae962bbib1] Anon (2024). IBTrACS. https://www.ncei.noaa.gov/products/international-best-track-archive.

[erlae962bbib2] Bates P D (2021). Combined modeling of US fluvial, pluvial, and coastal flood hazard under current and future climates. Water Resour. Res..

[erlae962bbib3] Bell B (2021). The ERA5 global reanalysis: preliminary extension to 1950. Q. J. R. Meteorol. Soc..

[erlae962bbib4] Benito I, Aerts J C J H, Ward P J, Eilander D, Muis S (2025). A multiscale modelling framework of coastal flooding events for global to local flood hazard assessments. Nat. Hazards Earth Syst. Sci..

[erlae962bbib5] Berger M J, George D L, LeVeque R J, Mandli K T (2011). The GeoClaw software for depth-averaged flows with adaptive refinement. Adv. Water Resour..

[erlae962bbib6] Dangendorf S, Sun Q, Maduwantha P, Wahl T, Marcos M, Marzeion B, Slangen A, Mitrovica J (2025). Human-driven sea-level rise has tripled the frequency of coastal sea-level extremes since 1900. Res. Square.

[erlae962bbib7] Dangendorf S, Sun Q, Wahl T, Thompson P, Mitrovica J X, Hamlington B (2024). Probabilistic reconstruction of sea-level changes and their causes since 1900. Earth Syst. Sci. Data.

[erlae962bbib8] Delforge D, Wathelet V, Below R, Lanfredi Sofia C, Tonnelier M, van Loenhout J A F, Speybroek N (2024). The EM-DAT emergency events database archive online. https://dataverse.uclouvain.be/dataset.xhtml?persistentId=doi:10.14428/DVN/I0LTPH.

[erlae962bbib9] Eilander D, Couasnon A, Leijnse T, Ikeuchi H, Yamazaki D, Muis S, Dullaart J, Haag A, Winsemius H C, Ward P J (2023a). A globally applicable framework for compound flood hazard modeling. Nat. Hazards Earth Syst. Sci..

[erlae962bbib10] Eilander D, Couasnon A, Sperna Weiland F C, Ligtvoet W, Bouwman A, Winsemius H C, Ward P J (2023b). Modeling compound flood risk and risk reduction using a globally applicable framework: a pilot in the Sofala province of Mozambique. Nat. Hazards Earth Syst. Sci..

[erlae962bbib11] Frieler K (2024). Scenario setup and forcing data for impact model evaluation and impact attribution within the third round of the inter-sectoral impact model intercomparison project (ISIMIP3a). Geosci. Model Dev..

[erlae962bbib12] Guzman O, Jiang H (2021). Global increase in tropical cyclone rain rate. Nat. Commun..

[erlae962bbib13] Hamlington B D, Bellas-Manley A, Willis J K, Fournier S, Vinogradova N, Nerem R S, Piecuch C G, Thompson P R, Kopp R (2024). The rate of global sea level rise doubled during the past three decades. Commun. Earth Environ..

[erlae962bbib14] Holland G J (1980). An analytic model of the wind and pressure profiles in hurricanes. Mon. Weather Rev..

[erlae962bbib15] Internal Displacement Monitoring Centre (2023). Global internal displacement database. https://www.internal-displacement.org/database/displacement-data/.

[erlae962bbib16] Islam R, Takagi H (2021). Statistical significance of tropical cyclone forward speed on storm surge generation: retrospective analysis of best track and tidal data in Japan. Georisk.

[erlae962bbib17] Kettner A J, Brakenridge G R, Schumann G J-P, Shen X, Schumann G J-P (2021). DFO—flood observatory. Earth Observation for Flood Applications.

[erlae962bbib18] Knapp K R, Kruk M C, Levinson D H, Diamond H J, Neumann C J (2010). The International Best Track Archive For Climate Stewardship (IBTrACS). Bull. Am. Meteorol. Soc..

[erlae962bbib19] Knutson T (2019). Tropical cyclones and climate change assessment: part I: detection and attribution. Bull. Am. Meteorol. Soc..

[erlae962bbib20] Knutson T (2020). Tropical cyclones and climate change assessment: part II: projected response to anthropogenic warming. Bull. Am. Meteorol. Soc..

[erlae962bbib21] Kossin J P (2018). A global slowdown of tropical-cyclone translation speed. Nature.

[erlae962bbib22] Kunze S, Strobl E A (2024). The global long-term effects of storm surge flooding on human settlements in coastal areas. Environ. Res. Lett..

[erlae962bbib23] Kvas A, Brockmann J M, Krauss S, Schubert T, Gruber T, Meyer U, Mayer-Gürr T, Schuh W-D, Jäggi A, Pail R (2021). GOCO06s—a satellite-only global gravity field model. Earth Syst. Sci. Data.

[erlae962bbib24] Lee W, Sun A Y, Scanlon B R, Dawson C (2024). Hindcasting compound pluvial, fluvial and coastal flooding during Hurricane harvey (2017) using Delft3D-FM. Nat. Hazards.

[erlae962bbib25] LeVeque R J, George D L, Berger M J (2011). Tsunami modelling with adaptively refined finite volume methods. Acta Numer..

[erlae962bbib26] Liang H, Zhou X (2022). Impact of tides and surges on fluvial floods in coastal regions. Remote Sens..

[erlae962bbib27] Liu G, Jiang S, Zheng M, Lin S, Kong Y, Zhan P (2025). A global ERA5-based tropical cyclone wind field dataset enhanced by integrated parametric correction methods. Sci. Data.

[erlae962bbib28] Lyard F H, Allain D J, Cancet M, Carrère L, Picot N (2021). FES2014 global ocean tide atlas: design and performance. Ocean Sci.

[erlae962bbib29] Mandli K T, Dawson C N (2014). Adaptive mesh refinement for storm surge. Ocean Modelling.

[erlae962bbib30] Masson-Delmotte V, Zhai P, Pirani A (2023). IPCC, 2021: summary for Policymakers. Climate Change 2021: The Physical Science Basis. Contribution of Working Group Ito the 6th Assessment Report of the Intergovernmental Panel on Climate Change.

[erlae962bbib31] Mechler R, Bouwer L M, Schinko T, Surminski S, Linnerooth-Bayer J (2018). Loss and Damage from Climate Change.

[erlae962bbib32] Mengel M (2026). Factual and counterfactual dataset of tropical cyclone-induced coastal flooding (v1.1). ISIMIP.

[erlae962bbib33] Mester B, Vogt T, Bryant S, Otto C, Frieler K, Schewe J (2023). Human displacements from tropical cyclone Idai attributable to climate change. Nat. Hazards Earth Syst. Sci..

[erlae962bbib34] Muis S, Apecechea M I, Dullaart J, de Lima Rego J, Madsen K S, Su J, Yan K, Verlaan M (2020). A high-resolution global dataset of extreme sea levels, tides, and storm surges, including future projections. Front. Mar. Sci..

[erlae962bbib35] Muis S, Verlaan M, Winsemius H C, Aerts J C J H, Ward P J (2016). A global reanalysis of storm surges and extreme sea levels. Nat. Commun..

[erlae962bbib36] Natural Earth (2025). Natural Earth—free vector and raster map data at 1:10m, 1:50m, and 1:110m scales. Nat. Earth.

[erlae962bbib37] Otto F E L (2023). Attribution of extreme events to climate change. Annu. Rev. Environ. Resour..

[erlae962bbib38] Paprotny D, ’t Hart C M P, Morales-Nápoles O (2024). Evolution of flood protection levels and flood vulnerability in Europe since 1950 estimated with vine-copula models. Nat. Hazards.

[erlae962bbib39] Pekel J-F, Cottam A, Gorelick N, Belward A S (2016). High-resolution mapping of global surface water and its long-term changes. Nature.

[erlae962bbib40] Sampson C C, Smith A M, Bates P D, Nea J C, Alfieri L, Freer J E (2015). A high-resolution global flood hazard model: a high-resolution global flood hazard model. Water Resour. Res..

[erlae962bbib41] Scussolini P, Aerts J C J H, Jongman B, Bouwer L M, Winsemius H C, de Moel H, Ward P J (2016). FLOPROS: an evolving global database of flood protection standards. Nat. Hazards Earth Syst. Sci..

[erlae962bbib42] Seeger K, Minderhoud P S J (2026). Sea level much higher than assumed in most coastal hazard assessments. Nature.

[erlae962bbib43] Seneviratne S I, Zhang X, Adnan M (2023). Weather and climate extreme events in a changing climate. Climate Change 2021: The Physical Science Basis. Contribution of Working Group I to the 6th Assessment Report of the Intergovernmental Panel on Climate Change.

[erlae962bbib44] Shen X, Anagnostou E N, Allen G H, Robert Brakenridge G, Kettner A J (2019). Near-real-time non-obstructed flood inundation mapping using synthetic aperture radar. Remote Sens. Environ..

[erlae962bbib45] Smiley K T, Noy I, Wehner M F, Frame D, Sampson C C, Wing O E J, Wing O E J (2022). Social inequalities in climate change-attributed impacts of Hurricane harvey. Nat. Commun..

[erlae962bbib46] Soci C (2024). The ERA5 global reanalysis from 1940 to 2022. Q. J. R. Meteorol. Soc..

[erlae962bbib47] Tellman B, Sullivan J A, Kuhn C, Kettner A J, Doyle C S, Brakenridge G R, Erickson T A, Slayback D A (2021). Satellite imaging reveals increased proportion of population exposed to floods. Nature.

[erlae962bbib48] Treu S, Muis S, Dangendorf S, Wahl T, Oelsmann J, Heinicke S, Frieler K, Mengel M (2024). Reconstruction of hourly coastal water levels and counterfactuals without sea level rise for impact attribution. Earth Syst. Sci. Data.

[erlae962bbib49] Tu S, Chan J C L, Xu J, Zhong Q, Zhou W, Zhang Y (2022). Increase in tropical cyclone rain rate with translation speed. Nat. Commun..

[erlae962bbib50] Vertegaal D M (2025). Climate and impact attribution of compound flooding induced by tropical cyclone idai in Mozambique. Nat. Hazards Earth Syst. Sci..

[erlae962bbib51] Vogt T, Treu S, Mengel M, Frieler K, Otto C (2024). Modeling surge dynamics improves coastal flood estimates in a global set of tropical cyclones. Commun. Earth Environ..

[erlae962bbib52] Wijetunge J J, Neluwala N G P B (2023). Compound flood hazard assessment and analysis due to tropical cyclone-induced storm surges, waves and precipitation: a case study for coastal lowlands of Kelani river basin in Sri Lanka. Nat. Hazards.

[erlae962bbib53] Yamaguchi M, Chan J C L, Moon I-J, Yoshida K, Mizuta R (2020). Global warming changes tropical cyclone translation speed. Nat. Commun..

[erlae962bbib54] Zhong Q, Chan J C L, Duan W, Tu S, Li J, Gan J, Ding R (2026a). Landfalling tropical cyclones accelerate due to land–sea thermal and roughness contrasts. Nat. Geosci..

[erlae962bbib55] Zhong Q, Gan J, Tu S, Toumi R, Chan J C L (2026b). Global increase in rain rate of tropical cyclones prior to landfall. Nat. Commun..

